# Optimal timing of ventriculoperitoneal shunt insertion relative to cranioplasty post-decompressive craniectomy: a frequentist network meta-analysis

**DOI:** 10.1007/s00701-026-06885-5

**Published:** 2026-05-12

**Authors:** Shaan Patel, Shiva A. Nischal, Kush M. Kale, Pious D. Patel, James S. Harrop, Jack Jallo, Srinivas K. Prasad

**Affiliations:** 1 Department of Neurological Surgery, Thomas Jefferson University Hospital, Philadelphia, PA USA; 2 Department of Physiology, Anatomy & Genetics, Medical Sciences Division, University of Oxford, Oxford, UK

**Keywords:** Cranioplasty, Cerebrospinal fluid, Decompressive craniectomy, Hydrocephalus, Network meta-analysis, Ventriculoperitoneal shunt

## Abstract

**Objective:**

Optimal timing of ventriculoperitoneal shunting (VPS) relative to cranioplasty (CP) after decompressive craniectomy remains controversial. VPS may be performed before, during, or after CP, but no consensus exists on which sequence minimizes complications. We performed a systematic review and network meta-analysis to compare the safety profiles of pre-, simultaneous, and post-CP shunting.

**Methods:**

We searched PubMed/MEDLINE, Embase, and CENTRAL from inception to October 2025. Eligible studies directly compared at least two timing strategies in patients undergoing both CP and VPS. Primary outcomes included overall complications, reoperation, infection, and intradural bleeding. Secondary outcomes included shunt-related and other procedure-specific adverse events. We used a frequentist random-effects network meta-analysis with Mantel–Haenszel pooling. Transitivity and consistency were assessed using design-by-treatment interaction modeling and node-splitting. Certainty of evidence was graded using CINeMA.

**Results:**

Ten observational studies met inclusion criteria, comprising 532 patients: 291 (54.7%) pre-CP VPS, 156 (29.3%) simultaneous CP-VPS, and 85 (16.0%) post-CP VPS. Simultaneous CP-VPS was associated with a significantly higher overall complication risk compared with post-CP VPS (RR 2.07; 95% CI 1.16–3.71; *P* < 0.05). Risk of reoperation (68/386, 17.6%), infection (62/532, 11.7%), and intradural bleeding (22/509, 2.8%) did not differ significantly across timing strategies. Secondary outcomes were infrequently reported and showed no statistically significant differences between groups. Inter-study heterogeneity and network inconsistency were low. Certainty of evidence for the main comparison (simultaneous versus post-CP VPS) was rated high.

**Conclusion:**

Simultaneous CP-VPS carried a higher composite complication risk than post-CP VPS, whereas staged strategies (VPS followed by CP or CP followed by VPS) demonstrated broadly comparable risk of infection, reoperation, and hemorrhage. These findings support a staged reconstruction paradigm in which CP is performed first to restore intracranial compliance and CSF hydrodynamics, with VPS reserved for persistent or progressive hydrocephalus rather than being routinely inserted at the time of CP.

**Supplementary Information:**

The online version contains supplementary material available at 10.1007/s00701-026-06885-5.

## Introduction

Cranioplasty (CP) restores the structural integrity of the skull and is routinely performed following decompressive craniectomy (DC). Beyond mechanical protection, CP has also been shown to promote neurological recovery [8, 36], optimize cerebral blood flow (CBF), and improve cerebrospinal fluid (CSF) circulation hydrodynamics [9, 11, 12, 45]. Physiologically, CP restores a closed intracranial system and normalizes intracranial pressure (ICP)-volume relationships, venous capacitance, and CSF dynamics [45].

A subset of patients treated with DC for malignant cerebral edema secondary to traumatic brain injury (TBI) or stroke develop hydrocephalus, with reported rates of 10–40% [49]. Importantly, whether DC causally increases the incidence of hydrocephalus, or whether reported shunt risk reflects selection bias and diagnostic ambiguity, remains debated. Patients undergoing DC represent the most severe end of the brain injury spectrum, and post-DC ventriculomegaly may reflect *ex vacuo* dilation or delayed cerebral re-expansion rather than true communicating hydrocephalus [6, 20]. Nonetheless, some patients will require insertion of a ventriculoperitoneal shunt (VPS) (~ 15% of cases post-DC) [26]. Yet, the optimal timing of VPS insertion relative to CP (i.e. before, during, or after) remains unresolved.

Each strategy carries distinct physiological and operative trade-offs. Pre-CP shunting offers early ICP control and ventricular decompression, but risks over-drainage, subdural collections, and paradoxical herniation [13, 48]. Post-CP shunting restores intracranial compliance first, but may delay definitive CSF diversion in patients with evolving hydrocephalus [49]. A single-stage simultaneous CP-VPS insertion streamlines care, yet several series have linked it to higher infectious and hemorrhagic complications [13, 32, 35], with some reporting advantages in selected settings [46].

The comparative literature is largely retrospective and heterogeneous in timing definitions, case-mix, implant materials, and valve types. For example, Gill et al. [13] reported fewer revisions with pre-CP shunting versus post-CP. Conversely, Zhang et al. [48] and Zheng et al. [49] observed greater complications with early diversion. An expert European consensus recently recommended a two-stage strategy, with CP first and VPS only if indicated, to minimize overtreatment and infection risk amidst diagnostic uncertainty [20]. A recent cohort study suggested slightly higher complications with pre-CP shunting but somewhat more implant failures when shunting followed CP, concluding that delaying VPS insertion after CP is generally preferable [41].

Beyond definitive CSF diversion, uncertainty persists regarding the optimal timing of CP within the post-DC timeline. While some studies suggest earlier CP (≤ 2 months) may reduce subsequent hydrocephalus risk [30], others report higher complications with early reconstruction (≤ 3 months) [40]. Temporary CSF management in this setting has primarily involved external ventricular or lumbar drainage, though comparative evidence remains limited [7]. These uncertainties underscore the need for a unified comparative synthesis.

To this end, we conducted a systematic review and frequentist network meta-analysis to compare pre-, simultaneous, and post-CP VPS. Our primary objective was to compare the risk of complication, reoperation, infection, and intradural bleeding across sequencing strategies.

## Methods

### Study design and reporting framework

We conducted this review in accordance with Cochrane guidelines [17] and reported it following the PRISMA-NMA extension (Supplementary Fig. 1) [19]. We registered a protocol a priori on PROSPERO (CRD420251171296).

### Data sources and search strategy

We comprehensively searched PubMed/MEDLINE, Embase, and Cochrane Central Register of Controlled Trials (CENTRAL) databases from inception to 29 October 2025 using predefined search terms (Supplementary Table 1). Study selection involved screening of titles-and-abstracts independently by two authors, followed by full-text evaluation for eligibility. Disagreements were resolved through panel discussion with a third reviewer.

### Eligibility criteria

We considered studies eligible for inclusion if they were: (i) randomized controlled trials or observational studies (case–control and cohort); (ii) directly compared at least two of pre-, simultaneous, or post-CP shunting strategies; (iii) enrolled adult or pediatric patients who had both procedures (CP and VPS insertion) performed; (iv) reported at least one of the pre-specified primary outcomes. We excluded studies that: (i) did not report any primary outcomes of interest; (ii) failed to distinguish pre-, simultaneous, and post-CP VPS outcome data; (iii) were editorials, letters, conference abstracts, case reports, or single-arm series; (iv) were non-English without available translation. There were no exclusion criteria applied on the basis of publication date. For overlapping cohorts, the most complete dataset was used unless different studies reported different outcomes.

### Outcomes of interest

The primary outcomes for this review included: (i) overall complications (defined as a composite of any post-operative adverse event reported by the original studies, including infectious, hemorrhagic, CSF-related, wound-related, or device-related complications, as classified by the reporting authors); (ii) reoperation (defined as any secondary surgery needed to treat a complication of the primary CP regardless of VPS insertion timing); (iii) infection (defined as surgical site infection (SSI) or CP implant infection); (iv) intradural bleeding (defined as any post-operative intracranial hemorrhage deep to the dura mater, including subdural hemorrhage (SDH), intracerebral hemorrhage (ICH), and intraventricular hemorrhage (IVH)). Of note, intradural bleeding was prioritized a priori given its direct relevance to ICP dynamics and CSF diversion physiology, whereas epidural hemorrhage (EDH) is predominantly influenced by CP-related factors such as implant fixation, dead space, and scalp tension; EDH was therefore analyzed as a secondary outcome. In addition, the inclusion of both a composite outcome and individual primary endpoints was intended to capture both the overall morbidity burden and specific clinically relevant complications, given variability in outcome reporting across studies.

Secondary outcomes of interest included: (i) VPS failure (defined as any mechanical malfunction of the shunt system, including obstruction or disconnection) or over-drainage phenomena (including siphoning or slit ventricle syndrome); (ii) VPS infection (defined as any infection localized to the proximal, distal, or reservoir shunt system); (iii) subdural effusion or hygroma (defined as any subdural fluid collection without evidence of acute hemorrhage); (iv) CSF leak (defined as any post-operative CSF egress through the incision or wound); (v) EDH (defined as any post-operative intracranial hemorrhage within the epidural space); (vi) bone flap resorption (defined as partial or complete resorption of autologous bone CP); (vii) syndrome of the trephined (defined as post-operative depression of the reconstructed cranial vault contour beneath the surrounding skull margins).

### Data extraction and synthesis

Data was independently extracted by two authors using a standardized form capturing study characteristics, demographics, timing definitions, valve type, CP material, and event counts for each outcome. Interventions were operationalized as timing strategies: pre- versus simultaneous versus post-CP VPS. We did not cluster by valve type or CP material (these were treated as effect modifiers, not separate nodes). For studies reporting multiple follow-up windows, we extracted data from the earliest post-procedural timepoint to prioritize temporal proximity between the timing strategy and observed complication, thereby reducing contamination from later events less directly attributable to sequencing (such as delayed shunt malfunction or long-term implant-related failure).

### Statistical analysis

Binary endpoints were analyzed using a frequentist random-effects Mantel–Haenszel network meta-analysis to estimate pooled risk ratios (RRs) with 95% confidence intervals (CIs). Pairwise and network analyses were performed in R v4.3.2 [31] (*R Core Team 2023*) using *netmeta* and *dmetar* packages [2]. Statistical significance was set at *P* < 0.05 (two-tailed). Inter-study heterogeneity was quantified using τ^2^ and I^2^ statistics via restricted maximum-likelihood estimation. Multi-arm trials were handled using shared-comparator variance splitting to avoid double-counting. Where studies reported adjusted comparative estimates by treatment timing, these were preferentially extracted, otherwise, unadjusted arm-level event counts were pooled. Zero-event studies were addressed using a uniform continuity correction of 0.5 for sparse data.

Network plots were generated for each endpoint to visualize network geometry and node connectivity. Ranking probabilities for each treatment were calculated using P-scores (0 to 1 scale, with a higher score indicating better overall performance of the competing treatment) [33]. Sensitivity analyses for statistically significant outcomes were performed via leave-one-out exclusion and exclusion of small study arms (n < 15) to assess robustness of findings.

### Assessment of transitivity and network consistency

The underlying transitivity assumption was evaluated qualitatively by comparing study and participant characteristics across treatment comparisons. Global inconsistency was assessed using a design-by-treatment interaction model, and local inconsistency was examined via node-splitting implemented using the *netsplit* function in *netmeta*, comparing direct and indirect evidence for each treatment contrast.

### Risk of bias and quality assessment

Two reviewers independently assessed study-level risk of bias using the Risk of Bias in Non-Randomized Studies of Interventions (ROBINS-I) [37]. Disagreements were resolved by consensus with a third reviewer. Certainty for each network estimate was summarized using the Confidence in Network Meta-Analysis (CINeMA) framework [28]. Small-study effects and publication bias were examined using comparison-adjusted funnel plots and Egger’s regression [34].

## Results

### Study selection and characteristics

**Fig. 1:**
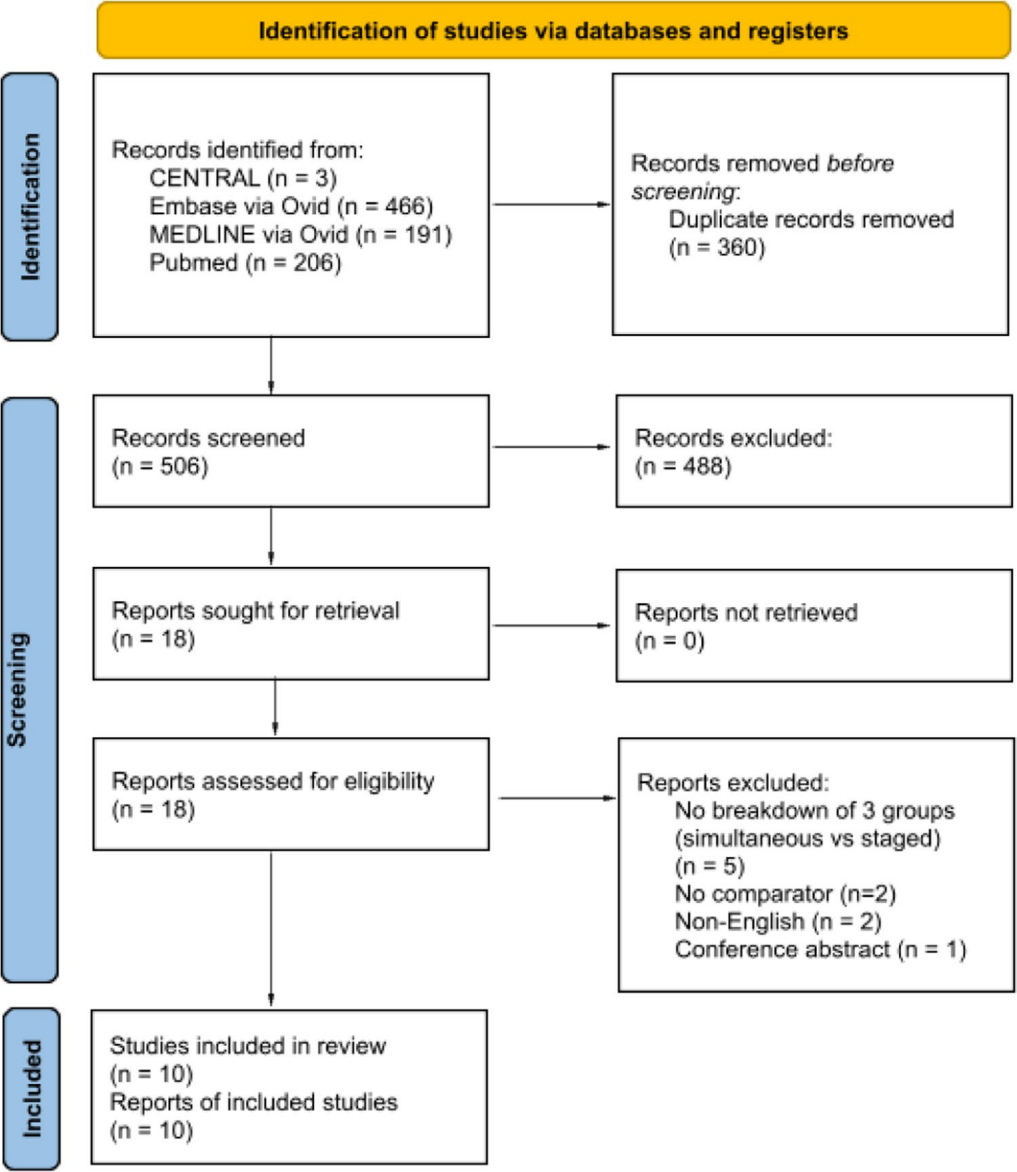
PRISMA flow diagram of study selection. Preferred Reporting Items for Systematic Reviews and Meta-Analyses (PRISMA) flow diagram outlining the study selection process. The number of records identified, screened, assessed for eligibility, and included in the final analysis are detailed at each stage

The initial search yielded 866 records, of which 18 full-text articles were assessed for eligibility (Fig. 1). Ten observational studies [4, 13, 16, 23, 29, 32, 35, 39, 41, 48] met the inclusion criteria, comprising a total of 532 patients who underwent both CP and VPS. Among these, 291 patients (54.7%) underwent pre-CP VPS, 156 (29.3%) underwent simultaneous CP-VPS, and 85 (16.0%) underwent post-CP VPS.

Mean age was 48.3 years for the pre-CP VPS group, 51.9 years for both the simultaneous and post-CP VPS groups. Proportion of male patients ranged from 40.9% to 100.0% across study arms. Mean follow-up ranged from 14.0 to 64.0 months. Across studies, the most common indications for DC were TBI and hemorrhagic stroke, with additional indications including SAH, cerebral infarction, ICH, IVH, and vascular lesions such as arteriovenous malformations (Table 1). The type and anatomical extent of DC (such as hemicraniectomy versus bifrontal craniectomy) were inconsistently reported and could not be analyzed comparatively.

**Table 1:** Study characteristics and patient demographics

Author (Year)	Country	Study design	Sample size (n)				Age (years) (mean ± SD or mean (range))			Sex (% male)	
			Total	Pre	Sim	Post	Pre	Sim	Post	Pre	Sim
Von der Brelie (2016)	Germany	RC	37	27	10	-	42.8 ± 17.9		-	NR	
Lin (2019)	Taiwan	RC	56	37	19	-	52.6 ± 15.6	57.5 ± 18.0	-	62.2	63.2
Heo (2014)	Korea	RC	51	19	32	-	55.3*	57.3*	-	68.4	56.2
Rosinski (2020)	USA	RC	40	22	18	-	45.0 ± 10.6	54.7 ± 10.1	-	40.9	16.7
Ting (2020)	Taiwan	RC	49	32	17	-	51.2 ± 17.5	57.4 ± 13.7	-	65.6	70.6
Schuss (2015)	Germany	RC	41	21	17	3	53.0 ± 18.0	52.0 ± 13.0	53.0 ± 18.0	42.0	41.0
Urvas (2015)	UK & Finland	RC	68	43	3	22	39.7 ± 22.5	41.2 ± 18.2	43.8 ± 24.8	46.5	59.1
Gill (2021)	Korea	RC	81	37	18	26	62.5 ± 12.4	60.9 ± 15.3	62.5 ± 12.4	57.1	33.3
Zhang (2022)	China	RC	84	40	22	24	39.2 ± 11.6	50.0 (43.0–57.0)	47.7 (18.2)	90.0	81.8
Oh (2008)	Korea	RC	23	13	-	10	41.0 (25.0–65.0)	-	41.0 (25.0–65.0)	60.0	-

Author (Year)	Country		Indication for DC (n)			Bifrontal index (n)			Degree of brain bulging (skin flap convexity) (n)		
		Post	Pre	Sim	Post	Pre	Sim	Post	Pre	Sim	Post
Von der Brelie (2016)	Germany	-	TBI Stroke*		-	NR		-	NR		-
Lin (2019)	Taiwan	-	TBI (24) ICH (2) SAH (4) Infarct (6) SDH (1)	TBI (15) ICH (2) Infarct (2)	-	0.39–0.46 (27)	0.36–0.43 (10)	-	Flaccid concave (11) Tense convex (26)	Flaccid concave (8) Tense convex (11)	-
Heo (2014)	Korea	-	TBI (12) SAH (12) ICH (6) Infarct (1) IVH (1)	TBI (12) SAH (12) ICH (6) Infarct (1) IVH (1)	-	≥ 0.40 (6) < 0.40 (13)	≥ 0.40 (19) < 0.40 (13)	-	Flaccid concave (3) Flaccid partial convex (4) Tense convex (12)	Flaccid concave (0) Flaccid partial convex (1) Tense convex (31)	-
Rosinski (2020)	USA	-	Vascular (18) Tumour/Infection (1) Other (3)	Vascular (18)	-	NR		-	NR		-
Ting (2020)	Taiwan	-	NR		-	> 0.50 (10) 0.40–0.50 (7) 0.30–0.40 (1)	> 0.50 (4) 0.40–0.50 (4) 0.30–0.40 (9)	-	Flaccid concave (1) Flaccid partial convex (7) Tense convex (24)	Flaccid concave (0) Flaccid partial convex (9) Tense convex (8)	-
Schuss (2015)	Germany	42.0	TBI (10) SAH (10) ICH (2) Infarct (2)	SAH (9) TBI (5) ICH (2) Infarct (1)	TBI (10) SAH (10) ICH (2) Infarct (2)	NR			NR		
Urvas (2015)	UK & Finland	100.0	Hemorrhage (17) TBI (12) Infarct (4) Other (10)	TBI (2) Other (1)	TBI (10) Hemorrhage (4) Infarct (3) Other (5)	NR			NR		
Gill (2021)	Korea	57.1	TBI (16) SAH (12) ICH (5) Infarct (2) AVM (1)	SAH (10) Infarct (3) TBI (2) ICH (2) AVM (1)	TBI (11) SAH (9) ICH (4) Infarct (2) AVM (1)	0.34 ± 0.03 (37)	0.37 ± 0.08 (18)	0.34 ± 0.03 (26)	Flaccid concave (5) Tense convex (32)	Flaccid concave (0) Tense convex (18)	Flaccid concave (3) Tense convex (23)
Zhang (2022)	China	70.8	TBI (20) ICH (14) SAH (6)	TBI (13) ICH (6) SAH (3)	TBI (17) ICH (5) SAH (2)	NR			Flaccid concave (1) Tense convex (39)	Flaccid concave (3) Tense convex (19)	Flaccid concave (9) Tense convex (15)
Oh (2008)	Korea	61.5	TBI (9) Infarct (2) SAH (2)	-	TBI (7) Infarct (2) SAH (1)	> 0.40 (13)	-	> 0.40 (10)	NR		

Baseline characteristics of included studies comparing pre-cranioplasty (CP) ventriculoperitoneal shunting (VPS), simultaneous CP-VPS, and post-CP VPS. Reported variables include study design, sample size, age, sex distribution, follow-up duration, indication for DC, radiographic indices used to define hydrocephalus (where available), and qualitative assessment of brain contour or bulging at the time of intervention. Data is reported at the study arm level where available. Not all variables were available for all timing strategies

Abbreviations & Symbols: *CP* Cranioplasty, *DC* Decompressive Craniectomy, *VPS* Ventriculoperitoneal Shunting, *Pre* VPS prior to CP, *Sim* VPS at the time of CP (i.e. simultaneous), *Post* VPS after CP, *RC* Retrospective Cohort, *DC* Decompressive Craniectomy, *TBI* Traumatic Brain Injury, *ICH* Intracerebral Hemorrhage, *IVH* Intraventricular Hemorrhage, *SAH* Subarachnoid Hemorrhage, *SDH* Subdural Hemorrhage, *AVM* Arteriovenous Malformation, *NR* Not Reported; *Cell where mean and/or standard deviation and/or range and/or n-number not reported

The average time between DC and subsequent surgery was 147.6 days when VPS was performed before CP, 98 days when it was performed simultaneously, and 88.2 days when it was performed after. Length of hospital stay was reported in a single study [32], with a mean stay of 41.6 ± 18.1 days in the pre-CP VPS group and 36.9 ± 12.5 days in the simultaneous CP-VPS group.

Where reported, the most common shunt types were programmable (235/290, 81%) and non-programmable valves (55/290, 19%). CP materials included autologous (74.7%, 195/261), titanium (18.4%, 48/261), and synthetic (6.9%, 18/261). Ventricular catheter entry sites were reported in 2 studies [32, 48] and included frontal and occipital approaches. Shunt laterality relative to DC location was reported in 1 study [13], with contralateral placement predominating (Table 2).

**Table 2:** Operative timing, technical variables, and device characteristics

Author (Year)	Cranial defect size (n)			Interval between DC and first subsequent operation (days) (mean ± SD or mean (range))			CP operative time (mins) (mean ± SD or mean (range))			VPS operative time (mins) (mean ± SD or mean (range))	
	Pre	Sim	Post	Pre	Sim	Post	Pre	Sim	Post	Pre	Sim
Von der Brelie (2016)	NR		-	NR		-	NR		-	NR	
Lin (2019)	NR		-	61.2 (39.6–82.9)	140.1 (84.1–196.1)	-	NR		-	NR	
Heo (2014)	< 50% hemi (1) ≥ 50% hemi (18)	< 50% hemi (6) ≥ 50% hemi (26)	-	118.8*	68.4*	-	NR		-	NR	
Rosinski (2020)	< 5 cm (1) 5–10 cm (4) > 10 cm (11) NR (6)	< 5 cm (1) 5–10 cm (7) > 10 cm (6) NR (4)	-	89.5 ± 72.8	50.9 ± 46.9	-	107.9 ± 43.3	219.0 ± 83.6	-	NR	
Ting (2020)	123.2 ± 26.0 cm^2^ (32)	117.9 ± 19.7 cm^2^ (17)	-	36.9 ± 21.1	52.4 ± 30.5	-	NR		-	NR	
Schuss (2015)	NR			107.0*	115.0*	107.0*	114.0 ± 38.0	139.0 ± 44.0	114.0 ± 38.0	57.0 ± 20.0	53.0 ± 15.0
Urvas (2015)	NR			249.2 ± 331.0	148.9 ± 115.0	63.8 ± 15.2	NR			NR	
Gill (2021)	< 50% hemi (7) ≥ 50% hemi (56)	< 50% hemi (4) ≥ 50% hemi (14)	< 50% hemi (7) ≥ 50% hemi (56)	115.4 ± 94.5	59.8 ± 29.4	93.9 ± 74.7	142.5 ± 39.7	180.0 ± 47.1	149.0 ± 52.6	62.1 ± 27.6	180.8 ± 47.1
Zhang (2022)	NR			158.0 (160.4–212.8)	103.3 (60.8–182.4)	68.9 (48.6–121.6)	NR			NR	
Oh (2008)	> 80 cm^3^	-	> 80 cm^3^	NR	-	NR	NR	-	NR	NR	-

Author (Year)	Cranial defect size (n)		VPS location (n)			Shunt type (n)			CP implant material (n)		
	Pre	Post	Pre	Sim	Post	Pre	Sim	Post	Pre	Sim	Post
Von der Brelie (2016)	NR	-	NR		-	NR		-	NR		-
Lin (2019)	NR	-	NR		-	NR		-	NR		-
Heo (2014)	< 50% hemi (1) ≥ 50% hemi (18)	-	NR		-	PV (50) Gravity-assisted (1)		-	NR		-
Rosinski (2020)	< 5 cm (1) 5–10 cm (4) > 10 cm (11) NR (6)	-	Right frontal (14) Left frontal (8)	Right frontal (10) Left frontal (8)	-	NR		-	Autologous (19) Synthetic (2) Titanium (1)	Autologous (18)	-
Ting (2020)	123.2 ± 26.0 cm^2^ (32)	-	NR		-	PV (9) NPV (23)	PV (6) NPV (11)	-	Autologous (32)	Autologous (16) Titanium (1)	-
Schuss (2015)	NR	57.0 ± 20.0	NR			NR			NR		
Urvas (2015)	NR		NR			NR			Autologous (8) Synthetic (16) Titanium (44)		
Gill (2021)	< 50% hemi (7) ≥ 50% hemi (56)	67.1 ± 32.1	Ipsilateral (7) Contralateral (30)	Ipsilateral (7) Contralateral (11)	Ipsilateral (8) Contralateral (18)	PV (81)			Autologous (79) Titanium (2)		
Zhang (2022)	NR		Frontal (21) Occipital (19)	Frontal (15) Occipital (7)	Frontal (11) Occipital (13)	PV (33) NPV (7)	PV (13) NPV (9)	PV (20) NPV (4)	NR		
Oh (2008)	> 80 cm^3^	NR	NR	-	NR	PV (13)	-	PV (10)	Autologous (13)	-	Autologous (10)

Operative and technical characteristics of included studies comparing pre-cranioplasty (CP) ventriculoperitoneal shunting (VPS), simultaneous CP-VPS, and post-CP VPS. Reported variables include cranial defect size, interval between DC and first subsequent procedure, cranioplasty (CP) operative time, VPS operative time, ventricular catheter entry site, shunt valve type, and CP implant material. Data is presented at the study arm level where available

Abbreviations & Symbols: *CP* Cranioplasty, *DC* Decompressive Craniectomy, *VPS* Ventriculoperitoneal Shunting, *Pre* VPS prior to CP, *Sim* VPS at the time of CP (i.e. simultaneous); Post, VPS after CP, *DC* Decompressive Craniectomy, *Hemi* Hemisphere, *PV* Programmable Valve, *NPV* Non-Programmable Valve, *NR* Not Reported; *Cell where mean and/or standard deviation and/or range not reported

### Network structure and geometry

**Fig. 2:**
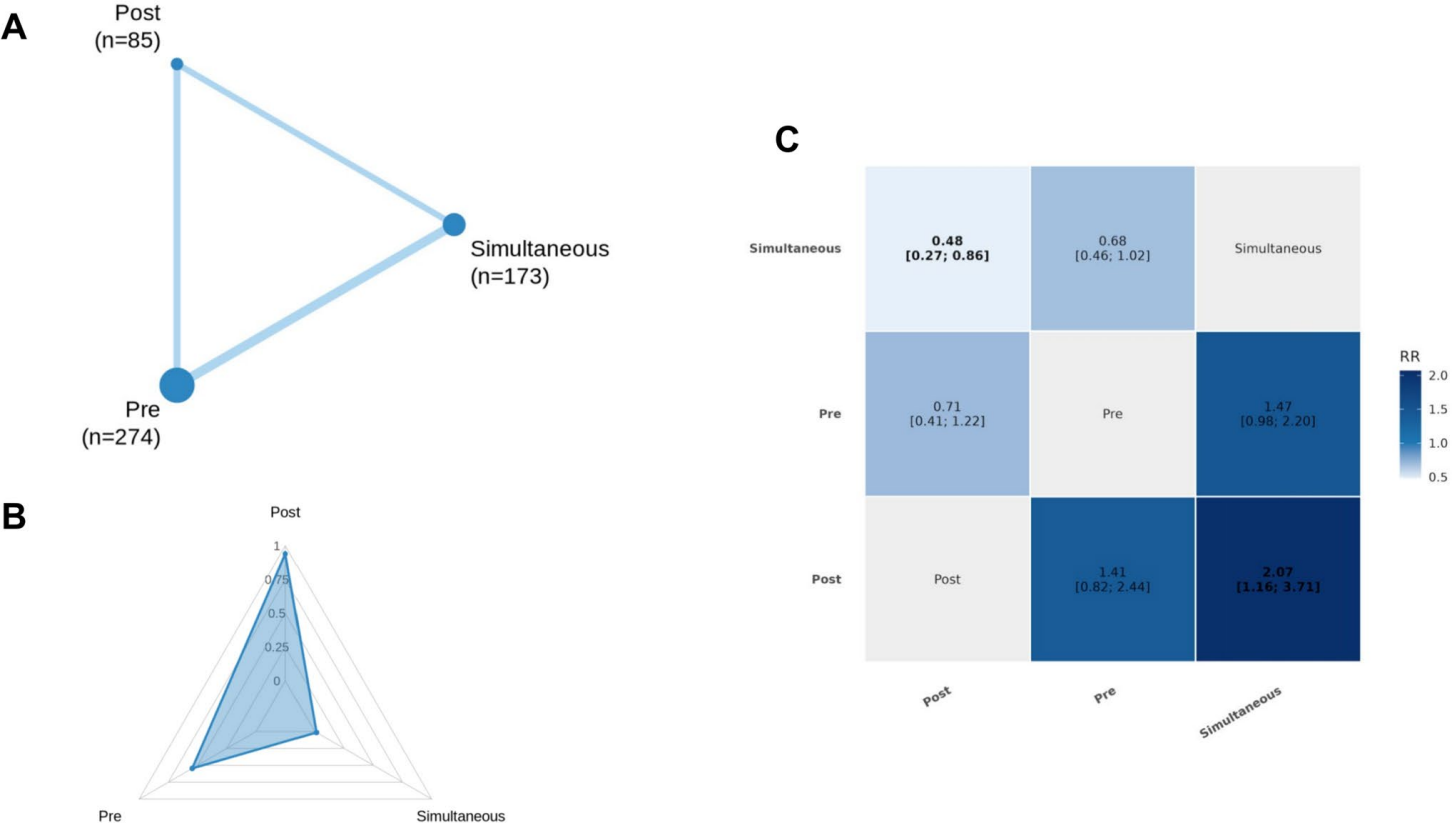
Network meta-analysis for overall complications. (**A**) Network plot showing the structure of available evidence across timing strategies. Nodes represent treatment strategies (pre-, simultaneous, and post-CP VPS), with node size proportional to the number of patients in each group and edge thickness reflecting the number of direct comparisons between strategies. (**B**) P score radar plot demonstrating the relative ranking of each strategy for overall complications (scale 0–1), where higher values indicate a greater probability of being the best-performing (lowest complication risk) strategy. (**C**) League table heatmap showing pooled pairwise risk ratios (RRs) with 95% confidence intervals for all comparisons. Values > 1 indicate higher complication risk in the row-defining strategy relative to the column-defining comparator, whereas values < 1 favor the row strategy. Color intensity reflects the magnitude of effect. Abbreviations: Pre = Ventriculoperitoneal shunt placement prior to cranioplasty. Simultaneous = Ventriculoperitoneal shunt placement at the time of cranioplasty. Post = Ventriculoperitoneal shunt placement after cranioplasty

**Fig. 3:**
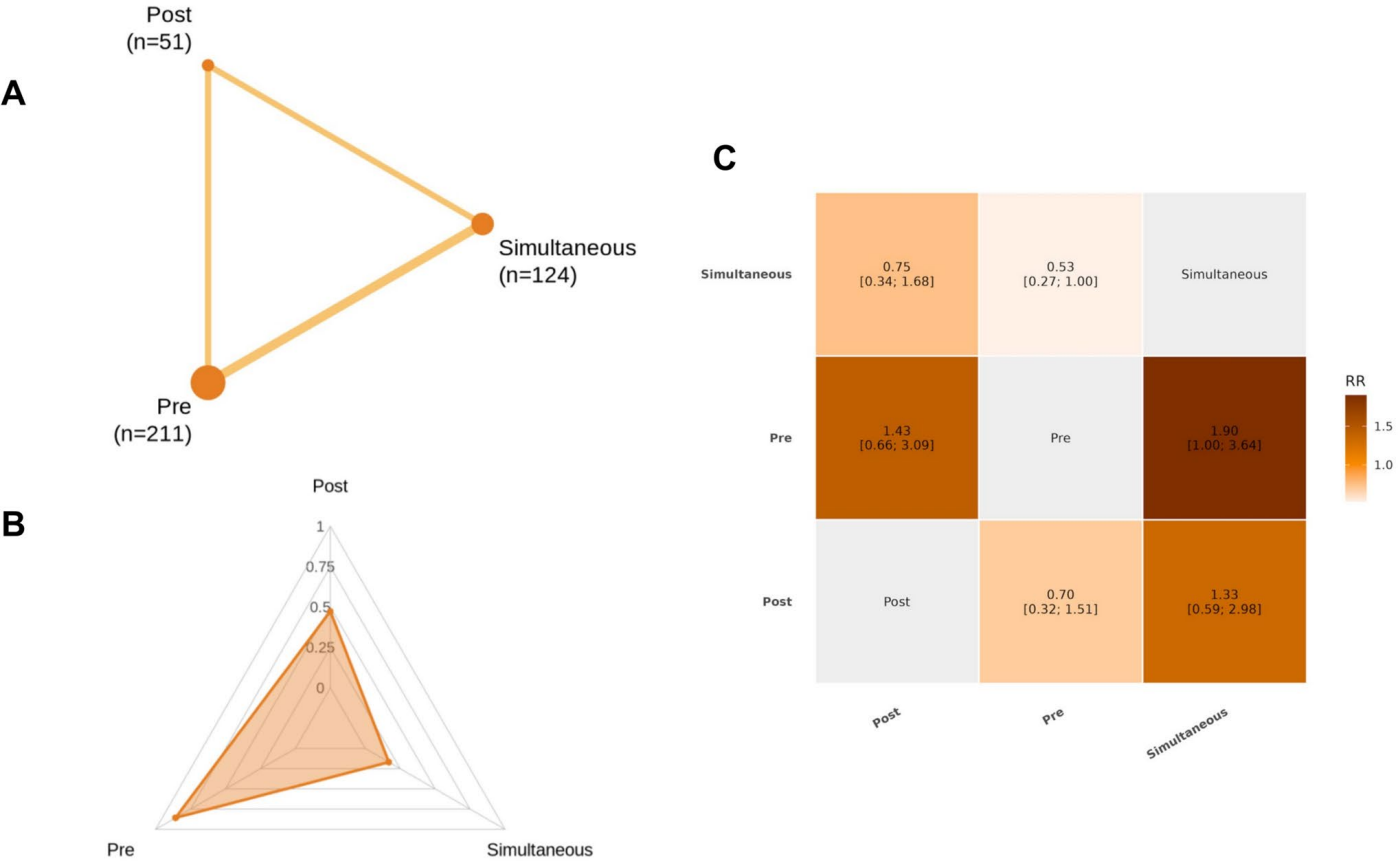
Network meta-analysis for reoperation. (**A**) Network plot showing the distribution of direct comparisons across timing strategy, with node size proportional to sample size and edge thickness reflecting comparative evidence. (**B**) P score radar plot demonstrating the relative ranking of each strategy for reoperation risk, where higher values indicate lower probability of requiring reoperation. (**C**) League table heatmap showing pooled pairwise risk ratios (RRs) with 95% confidence intervals. Values > 1 indicate higher reoperation risk in the row-defining strategy relative to the column-defining comparator, whereas values < 1 favor the row strategy. Color intensity reflects the magnitude of effect. Abbreviations: Pre = Ventriculoperitoneal shunt placement prior to cranioplasty. Simultaneous = Ventriculoperitoneal shunt placement at the time of cranioplasty. Post = Ventriculoperitoneal shunt placement after cranioplasty

**Fig. 4:**
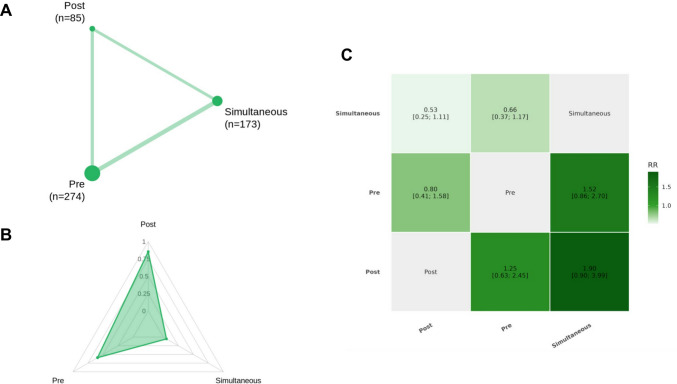
Network meta-analysis for surgical site infection. (**A**) Network plot showing the geometry of evidence across timing strategies, with node size and edge thickness reflecting study size and number of direct comparisons, respectively. (**B**) P score radar plot demonstrating the relative ranking of each strategy for infection risk, where higher values indicate lower infection risk. (**C**) League table heatmap showing pooled pairwise risk ratios (RRs) with 95% confidence intervals. Values > 1 indicate higher infection risk in the row-defining strategy relative to the column-defining comparator, whereas values < 1 favor the row strategy. Color intensity reflects the magnitude of effect. Abbreviations: Pre = Ventriculoperitoneal shunt placement prior to cranioplasty. Simultaneous = Ventriculoperitoneal shunt placement at the time of cranioplasty. Post = Ventriculoperitoneal shunt placement after cranioplasty

**Fig. 5:**
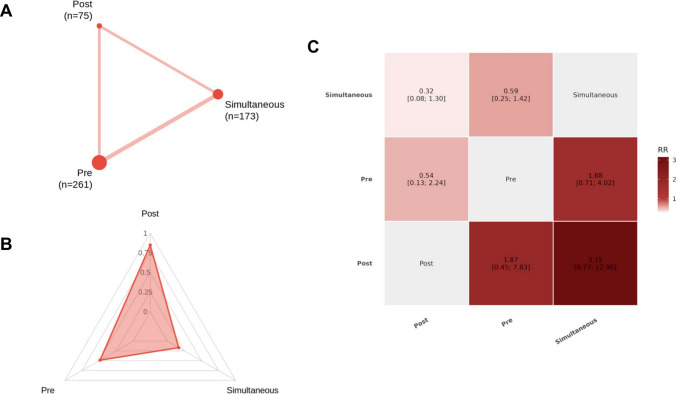
Network meta-analysis for intradural bleeding. (**A**) Network plot showing direct and indirect comparisons between timing strategies, with node size proportional to sample size and edge thickness reflecting available direct evidence. (**B**) P score radar plot demonstrating the relative ranking of each strategy for intradural bleeding risk, where higher values indicate lower bleeding risk. (**C**) League table heatmap showing pooled pairwise risk ratios (RRs) with 95% confidence intervals. Values > 1 indicate higher bleeding risk in the row-defining strategy relative to the column-defining comparator, whereas values < 1 favor the row strategy. Color intensity reflects the magnitude of effect. Abbreviations: Pre = Ventriculoperitoneal shunt placement prior to cranioplasty. Simultaneous = Ventriculoperitoneal shunt placement at the time of cranioplasty. Post = Ventriculoperitoneal shunt placement after cranioplasty

Each endpoint formed a tri-nodal network representing pre-CP, simultaneous, and post-CP VPS strategies (Fig. 2A, 3A, 4A, 5A). Direct evidence was most abundant for pre-CP versus post-CP shunting, and pre-CP versus simultaneous CP shunting comparisons, whereas simultaneous CP versus post-CP shunting contrasts were informed mainly by indirect evidence. Post-CP shunting arms contributed smaller sample sizes (85 patients) relative to pre-CP shunting (291 patients), producing modest network imbalance. Complete league tables for all primary outcomes are also presented (Fig. 2C, 3C, 4C, 5C).

### Overall complications

Overall complication events were reported in 10 studies [4, 13, 16, 23, 29, 32, 35, 39, 41, 48] (532 patients) and occurred in 25.1% (73/291) of the pre-CP VPS group, 38.5% (60/156) of the simultaneous CP-VPS group, and 23.5% (20/85) of the post-CP VPS group (Table 3). Simultaneous CP-VPS was associated with a significantly higher risk of complications compared with post-CP VPS (RR 2.07; 95% CI: 1.16–3.71; *P* < 0.05; I^2^ = 73%) (Fig. 2C). No significant differences were observed between pre-CP versus post-CP VPS, or pre-CP versus simultaneous CP shunting, however, pre- versus simultaneous CP shunting trended toward statistical significance (RR 0.68; 95% CI: 0.46–1.02). Leave-one-out and small-arm exclusions yielded stable estimates, confirming robustness of the main effect.

**Table 3:** Post-operative complications by timing strategy

Author (Year)	Pre-CP VPS		Simultaneous CP-VPS		Post-CP VPS	
	Complications (n)	Total (n)	Complications (n)	Total (n)	Complications (n)	Total (n)
Von der Brelie (2016)	Aseptic necrosis (3) Empyema (1)	4/27	Aseptic necrosis (3) Empyema (3)	6/10	-	-
Lin (2019)	Infection—surgical site (4) Infection—shunt (2)	5/37	CSF over-drainage (2) Infection—surgical site (1)	3/19	-	-
Heo (2014)	Subdural effusion (2) Infection—surgical site (1) Bleed—SDH (1)	4/19	Subdural effusion (8) Infection—surgical site & CP (6) Bleed—SDH (2) Bleed—ICH (1) Bleed—EDH (1)	18/32	-	-
Rosinski (2020)	Infection—surgical site (3) Infection—CP (1) Bone flap resorption (1)	8/22	Infection—shunt (2) Infection—surgical site (1) Infection—CP (1) Bleed—SDH (1) CSF leak (1)	6/18	-	-
Ting (2020)	Bleed—ICH (2) Infection—CP (1) Subdural effusion (1)	3/32	Infection—shunt (2) Subdural effusion (2) Infection—CP (1) Bleed—ICH (1)	5/17	-	-
Schuss (2015)	Bleed—unspecified (1) Hygroma (1)	2/21	Infection—unspecified (7) Bleed—unspecified (1)	8/17	Hygroma (1)	1/3
Urvas (2015)	Infection—surgical site (11) CSF leak (3) Bleed—unspecified (2) Wound dehiscence (2) Poor cosmetic outcome (1) Bone flap resorption (1)	20/43	Bleed—unspecified (2)	2/3	Infection—surgical site (4) Bleed—unspecified (1) Wound dehiscence (1) Poor cosmetic outcome (1) Bone flap resorption (1)	9/22
Gill (2021)	Infection—surgical site (2) Infection—shunt (1)	3/37	Bleed—EDH (2) Abscess—epidural (1) Abscess—cerebral (1) Bleed—IVH (1) Pneumocephalus (1)	6/18	Infection—surgical site (3) Infection—shunt (1)	4/26
Zhang (2022)	Syndrome of the trephined (6) Hygroma (4) Bleed—unspecified (3) Shunt obstruction (2) Seizure (2) Infection—surgical site (1)	15/40	Infection—surgical site (5) Hygroma (3) Seizure (1)	8/22	Infection—surgical site (2) Shunt obstruction (1)	3/24
Oh (2008)	Bleed—EDH (2) Subdural effusion (2) Slit ventricle (2) Seizure (2) Infection—unspecified (1)	9/13	-	-	Bleed—EDH (1) Subdural effusion (1) Ventricular dilatation (1)	3/10

Post-operative complications reported in included studies, stratified by ventriculoperitoneal shunting (VPS) relative to cranioplasty (CP): pre-CP VPS, simultaneous CP-VPS, and post-CP VPS. Complications are listed as reported in the original studies and include infectious, hemorrhagic, cerebrospinal fluid (CSF)-related, and reconstruction-related complications. For each study and timing strategy the number of patients experiencing at least one complication is presented alongside the total number of patients in the treatment arm

Abbreviations & Symbols: *CP* Cranioplasty, *VPS* Ventriculoperitoneal Shunting, *CSF* Cerebrospinal Fluid, *EDH* Extradural Hemorrhage, *ICH* Intracerebral Hemorrhage, *SDH* Subdural Hemorrhage

### Reoperation

Reoperation events were reported in 7 studies [13, 16, 23, 32, 35, 39, 41] (386 patients) and occurred in 12.8% (27/211) of the pre-CP VPS group, 23.4% (29/124) of the simultaneous CP-VPS group, and 23.5% (12/51) of the post-CP VPS group. There were no statistically significant differences between any pairwise comparisons of timing strategy, however, pre-CP versus simultaneous CP VPS trended toward statistical significance (RR 0.53; 95% CI: 0.27–1.00) (Fig. 3C).

### Infection

Infection events (CP or SSI) were reported in 10 studies [4, 13, 16, 23, 29, 32, 35, 39, 41, 48] (532 patients) and occurred in 62 patients (11.7%). There were no statistically significant differences between any pairwise comparisons of timing strategies (Fig. 4C).

### Intracranial bleeding

Intradural bleeding events (ICH, IVH, or SDH) were reported in 9 studies [4, 13, 16, 23, 32, 35, 39, 41, 48] (509 patients) and occurred in 22 patients (2.8%). There were no statistically significant differences between any pairwise comparisons of timing strategies (Fig. 5C). EDH events were reported in 4 studies [4, 13, 16, 48] (192 patients) and occurred in 2/96 (2.1%) of the pre-CP VPS group, 3/60 (5%) of the simultaneous CP-VPS group, and 1/36 (2.8%) of the post-CP VPS group.

### Shunt-related complications

VPS failure or over-drainage events were reported in 6 studies [4, 16, 23, 29, 41, 48] (321 patients) and occurred in 17/179 (9.5%) of the pre-CP VPS group, 10/86 (11.6%) of the simultaneous CP-VPS group, and 9/56 (16.1%) of the post-CP VPS group.

VPS infection events were reported in 5 studies [13, 16, 23, 32, 39] (277 patients) and occurred in 5/147 (3.4%) of the pre-CP VPS group, 5/104 (4.8%) of the simultaneous CP-VPS group, and 1/26 (3.8%) of the post-CP VPS group.

### CSF-related complications

Subdural effusion or hygroma events were reported in 6 studies [4, 16, 29, 35, 39, 48] (287 patients) and occurred in 25 patients (8.7%). There were no statistically significant differences between any pairwise comparisons of timing strategies (Supplementary Fig. 2).

CSF leak events were reported in 2 studies [32, 41] (108 patients) and occurred in 3/65 (4.6%) of the pre-CP VPS group, 1/21 (4.8%) of the simultaneous CP-VPS group, and 0/22 (0.0%) of the post-CP VPS group.

### Reconstruction-related complications

Bone flap resorption events were reported in 3 studies [4, 32, 41] (155 patients) and occurred in 5/92 (5.4%) of the pre-CP VPS group, 4/31 (12.9%) of the simultaneous CP-VPS group, and 0/22 (0.0%) of the post-CP VPS group.

Syndrome of the trephined events were reported in 3 studies [4, 39, 48] (172 patients) and occurred in 6/99 (6.0%) of the pre-CP VPS group, 2/49 (4.1%) of the simultaneous CP-VPS group, and 0/24 (0.0%) of the post-CP VPS group.

### Ranking of treatment strategies

Across endpoints, post-CP VPS consistently achieved the highest P-scores, ranking best for overall complications (0.94), infection (0.85), and intradural bleeding (0.85). Pre-CP VPS ranked highest for reoperation (0.86), whereas simultaneous CP-VPS shunting ranked lowest across all outcomes (Fig. 2B, 3B, 4B, 5B). The hierarchy of the tested treatment strategies is also provided in rankograms (Supplementary Fig. 3).

### Network consistency and heterogeneity

All network models converged well. Using the design-by-treatment interaction inconsistency model with restricted maximum likelihood estimation, we found no evidence of global inconsistency across primary outcomes (overall complications *P* = 0.78; reoperation *P* = 0.65; infection *P* = 0.41; intradural bleeding *P* = 0.36). Node-splitting analysis similarly demonstrated low statistical inconsistency between direct and indirect evidence, supporting the robustness of the network estimates (Supplementary Fig. 4). The proportions of direct versus indirect evidence contributing to each comparison in the network are also presented in node-split plots (Supplementary Fig. 5). Between-study heterogeneity was low across primary outcomes (overall complications I^2^ = 0.0%; reoperation I^2^ = 0.0%; infection I^2^ = 5.6%; intradural bleeding I^2^ = 7.2%).

### Risk of bias, publication bias, and certainty of evidence

Visual inspection of funnel plots across primary outcomes demonstrated symmetry, with all studies contained within the 95% CI pyramidal lines (Supplementary Fig. 6), suggesting low likelihood of publication bias. Egger’s regression test showed no evidence of small-study effects for all measured outcomes (*p* > 0.05). Based on the ROBINS-I tool, five studies were judged low risk of bias, three moderate, and two serious (Supplementary Table 2), with confounding as the most frequent source of bias. Based on the CINeMA framework, certainty for all timing comparisons on overall complications was high, within minimal downgrading due to imprecision, within-study bias, and heterogeneity (Supplementary Fig. 7).

## Discussion

The optimal sequencing of VPS placement and CP after DC remains a core and contentious issue in neurosurgical cranial reconstruction. Across ten observational studies encompassing 532 patients, this network meta-analysis represents the most comprehensive and statistically robust synthesis to date comparing pre-CP VPS, simultaneous CP-VPS, and post-CP VPS strategies. Our findings indicate that simultaneous CP-VPS is associated with a significantly higher risk of overall complications relative to post-CP VPS, while reoperation, infection, and intradural bleeding risk did not differ significantly between approaches. Collectively, these findings suggest that although simultaneous intervention may reduce operative sessions, it carries a measurable trade-off in post-operative morbidity. Importantly, although post-CP VPS demonstrated the most favorable ranking across outcomes, no statistically significant differences were observed between pre-CP and post-CP VPS for primary endpoints. This suggests that both staged strategies are acceptable, and that the observed ranking should be interpreted as a trend toward lower complication risk rather than definitive superiority.

### Mechanistic interpretation: restoring intracranial compliance and CSF homeostasis

DC alters cranial biomechanics, exposing the brain to a pathological pressure gradient between the intracranial compartment and atmosphere. CP reconstitutes the closed cranial system, restoring intracranial compliance and normalizing CBF and CSF hydrodynamics via enhanced venous capacitance and buffering CSF pulsatility [9, 11, 12, 15, 22, 42, 45]. However, in a subset of patients, the chronic distortion of CSF pathways post-DC culminates in hydrocephalus, with shunt placement required to re-establish CSF diversion [26, 49]. Although the trans-calvarial pressure gradient is present immediately after DC, its clinical expression is often delayed [18]. In the early post-DC phase, residual cerebral edema and increased parenchymal turgor partially counterbalance atmospheric forces. As edema resolves over weeks to months, progressive loss of intracranial volume buffering, venous collapse, and CSF redistribution can unmask the pressure differential, explaining the delayed onset of syndrome of the trephined phenomena in some patients [14].

The timing of shunt insertion relative to CP therefore directly influences intracranial fluid dynamics. Simultaneous CP and VPS may produce competing pressure gradients where acute reduction in intracranial elastance from CP coupled with shunt-driven CSF egress can transiently over-drain the ventricular system, predisposing to subdural effusions, epidural collections, or bone flap separation. Conversely, premature VPS before cranial reconstruction can perpetuate syndrome of the trephined, where continuous CSF diversion exacerbates the inward atmospheric gradient across the craniectomy window. In contrast, delayed VPS insertion may allow for gradual re-equilibration of CSF pathways and venous compliance before shunt dependence is declared.

These biomechanical frameworks are consistent with our finding of lower complication risk with post-CP shunting, aligning with recent consensus recommending staged procedures [20].

### Simultaneous intervention: convenience versus complication

Proponents of a single-stage strategy argue that it reduces anesthetic exposure, hospital stay, and resource utilization. Indeed, some institutional series described comparable safety when both operations were performed concurrently under optimized conditions [13, 32, 48]. Nevertheless, our pooled results and others [35] indicated that combining procedures substantially increased overall complication risk, particularly for CSF-related sequelae and hematoma formation. This excess risk likely reflected the convergence of procedural and physiological stressors inherent to a combined operation. Introduction of prosthetic hardware within a freshly reconstructed field increases tissue handling, dead-space formation, and CSF soft-tissue interface disruption. All these factors predispose to wound complications and device malfunction.

At a pathophysiological level, concurrent CP and VPS insertion may destabilize post-DC equilibrium between parenchymal turgor and subdural compliance. Following large-volume decompression, the brain may remain edematous, and early CP can transiently compress the swollen parenchyma. Even when CP is delayed, after the resolution of overt edema, VPS-induced ventricular decompression before compensatory venous adaptation may facilitate subdural hygroma or intradural bleeding. Waziri et al. [43] demonstrated that early CP alone can restore CSF dynamics and reduce long-term shunt dependence. This risk is compounded by impaired autoregulation and transient hyperemia in the early post-DC period [5, 24]. Experimental studies demonstrate that impaired CO_2_ reactivity and loss of vasomotor tone during ICP perturbations exacerbate subdural fluid shifts and venous congestion [10, 47]. These considerations argue against simultaneous reconstruction and diversion in patients with incomplete brain re-expansion or evolving intracranial compliance.

Furthermore, simultaneous surgery may increase theoretical contamination via shunt tunnelling across fresh CP tissue planes although infection risk did not differ significantly in our analysis.

### Pre-cranioplasty shunting: hydrocephalus control at a cost

Insertion of a shunt before CP remains common practice in patients with overt ventriculomegaly, progressive neurological decline, or poor brain re-expansion. However, poor brain re-expansion and syndrome of the trephined phenotypes represent relative contraindications to shunt placement due to risk of negative pressure complications. Excessive CSF drainage without cranial coverage can exacerbate paradoxical herniation or syndrome of the trephined by accentuating the trans-calvarial pressure gradient. Negative pressure gradients alter transmural venous and arteriolar tension, reducing cerebral venous outflow and microvascular perfusion [3, 44]. This reflects pressure-passive physiology [15]. Our findings align with prior studies [13, 49] reporting higher incidences of subdural effusion and cortical collapse with pre-CP shunting.

Pre-CP shunting may therefore be justified when hydrocephalus is symptomatic, progressive, and combined with careful post-operative monitoring. Limited data [9, 10, 27] suggests a mean interval of 10.1 weeks between VPS and CP in staged approaches.

### Post-cranioplasty shunting: physiological rationale and clinical support

Post-CP VPS ranked highest across endpoints and demonstrated the lowest complication risk. Mechanistically, performing CP first restores intracranial volume buffering, normalizes transmantle gradients, and stabilizes CSF and venous outflow dynamics. This may allow spontaneous resolution of borderline ventriculomegaly, distinguishing bona fide communicating hydrocephalus from transient ventricular dilation due to loss of skull rigidity [21, 25, 38]. This selection effect may also explain the higher risk of shunt failure or over-drainage in the post-CP VPS group, as patients proceeding to delayed VPS likely represent a cohort with definitive CSF diversion dependency rather than transient ventriculomegaly. Clinical series suggest that CP alone obviates shunt placement in up to one-third of patients [46]. Chibbaro et al. [6] reported stabilization of CSF hydrodynamics in 80% of cases, with only 17% requiring VPS. Similarly, early CP (< 2 months) has been associated with improved cortical perfusion and reduced long-term hydrocephalus risk [30].

Delayed shunting also enables more accurate diagnosis and valve titration. Conventional indices such as Evans’ ratio are unreliable in decompressed skulls [20] and clinical evolution remains central to decision-making. Cleaner operative planes during delayed VPS may further reduce risk of shunt tract contamination and wound tension, potentially contributing to lower complication risk.

### Valve technology, laterality, and secondary considerations

In our pooled cohort, programmable valves were used in over 80% of cases, reflecting modern practice and allowing post-operative adjustment to evolving intracranial compliance [1, 20]. This is particularly valuable in staged reconstruction, where CSF dynamics evolve between CP and subsequent VPS insertion. Shunt laterality appeared to have minimal impact on outcomes, with ipsilateral placement offering logistical advantages without compromising efficacy [46]. Finally, the long-term integrity of autologous bone flaps may be affected by chronic shunt diversion, with VPS identified as a risk factor in prior studies [27].

### Network interpretation and comparative context

Our network incorporated both direct and indirect evidence and demonstrated low inconsistency. While rankings should be interpreted cautiously, the consistent signal favoring post-CP shunting across analyses supports a true underlying effect. The absence of significant differences between pre- and post-CP shunting strategies suggests that staged approaches are broadly comparable, allowing individualized decision-making. These findings align with European consensus recommendations [20] and earlier single-center observations [13, 35, 48] emphasizing avoidance of simultaneous procedures. Notably, although reoperation events were numerically higher in the post-CP VPS group, these differences were not statistically significant and were based on limited sample sizes, and therefore should be interpreted cautiously in the context of the overall complication profile.

### Methodological considerations and future directions

All included studies were observational, introducing potential confounding by indication. Sample sizes were modest, limiting subgroup analyses by valve type, implant material, or timing interval thresholds. Outcome definitions varied, although sensitivity analyses supported robustness. The composite outcome of “overall complications” reflects heterogeneous definitions across included studies and should be interpreted as a pragmatic measure of morbidity rather than a standardized endpoint. Some network comparisons, particularly simultaneous versus post-CP shunting, relied on indirect evidence. Secondary outcomes were infrequently reported and should be interpreted as exploratory signals. Comparability between timing cohorts may have been limited by heterogeneity in the clinical indication for staging. Reporting of hydrocephalus status at the time of CP was inconsistent, introducing potential misclassification and limiting assessment of transitivity. Peri-CP CSF management strategies were also incompletely reported.

Future work should prioritize prospective multicenter studies with standardized definitions of hydrocephalus, consistent follow-up durations, and stratification by implant material and valve type. Integration of mechanistic biomarkers, such as perfusion imaging, ICP waveform analysis, and CSF flow mapping, could clarify causal links between intracranial physiology and post-operative outcomes. Objective compliance metrics would also help determine which patients benefit from delayed observation versus immediate diversion. Moreover, exploring how programmable valve technology can mitigate negative-pressure gradients in pre-CP shunting could refine individualized management algorithms. These approaches may enable more precise, physiology-driven decision-making.

## Conclusion

This network meta-analysis demonstrates that simultaneous CP-VPS is associated with higher overall complication risk compared with post-CP VPS, while staged strategies show comparable risk of infection, hemorrhage, and reoperation. These findings support a staged reconstruction paradigm in which both pre- and post-CP VPS represent acceptable strategies, with post-CP VPS demonstrating a consistent trend toward lower complication risk. Future prospective studies are required to define optimal timing strategies.

## Supplementary Information

Below is the link to the electronic supplementary material.

Supplementary file1 (DOCX 9736 KB)

## Data Availability

Not applicable.

## Abbreviations

CBF: Cerebral Blood Flow
CINeMA: Confidence in Network Meta-Analysis
CI: Confidence Interval
CP: Cranioplasty
CSF: Cerebrospinal fluid
DC: Decompressive Craniectomy
ICH: Intracerebral Hemorrhage
ICP: Intracranial Pressure
IVH: Intraventricular Hemorrhage
ROBINS-I: Risk of Bias in Non-Randomized Studies of—Interventions
RR: Risk Ratio
SAH: Subarachnoid Hemorrhage
TBI: Traumatic Brain Injury
VPS: Ventriculoperitoneal Shunt(ing)
SSI: Surgical Site Infection
